# The Glomeromycota in the Neotropics

**DOI:** 10.3389/fmicb.2020.553679

**Published:** 2021-01-12

**Authors:** Sidney Luiz Stürmer, Karl Kemmelmeier

**Affiliations:** Laboratory of Mycorrhiza, Department of Ciências Naturais, Universidade Regional de Blumenau (FURB), Blumenau, Brazil

**Keywords:** biogeography, biomes, South America, arbuscular mycorrhizal fungi, species distribution, Central America

## Abstract

Arbuscular mycorrhizal fungi (AMF—Glomeromycota) are a group of soil fungi with a widespread occurrence in terrestrial ecosystems where they play important roles that influence plant growth and ecosystem processes. The aim of this paper is to reveal AMF distribution in the Neotropics based on an extensive biogeography database with literature data from the last five decades. All four orders and 11 families were reported in the Neotropics. 221 species (69% of the total number of species for the phylum) were registered in the Neotropics pertaining to 37 genera. *Acaulospora*, *Glomus*, *Scutellospora*, and *Funneliformis* were the most speciose genera and represented by 47, 29, 15, and 13 species, respectively. Seventy-six species were originally described from Neotropics, which represents 24% of the total diversity of Glomeromycota. The most representative families were Gigasporaceae, Ambisporaceae, and Acaulosporaceae with 89%, 80%, and 79% of species within each family detected in the Neotropics, respectively. AMF were detected in 11 biomes and 52 ecological regions in 19 countries. Biomes with the largest number of species were Tropical and Subtropical Moist Forests (186 species), Tropical and Subtropical Dry Broadleaf forests (127 species), and Tropical and Subtropical Grasslands (124 species), and Jaccard’s similarity among them was 53–57%. Mean annual temperature and precipitation were not correlated with total AMF species richness. The Neotropics biomes shelter a large amount of the total diversity of Glomeromycota and studies of occurrence of these fungi should be encouraged considering their importance in maintaining terrestrial ecosystems.

## Introduction

Fungi in the phylum Glomeromycota include the arbuscular mycorrhizal fungi (AMF) which are associated with vascular plants and thalloid bryophytes (Brundrett and Tedersoo, 2018) and *Geosiphon pyriformis* associated with the cyanobacteria *Nostoc* (Schussler et al., 1994). Fossil evidences place the origin of AMF for at least 400 million years in the Devonian period where they were associated with the first vascular land plants from the Rhynie chert formation (Remy et al., 1994; Dotzler et al., 2006, 2009). Considering the lack of host specificity and the long geological time they have to disperse around the globe, it is not surprising that AMF are widely widespread in terrestrial ecosystems forming the arbuscular mycorrhizal association with 72% of plants species (Brundrett and Tedersoo, 2018) in all biogeographic realms and biomes (Stürmer et al., 2018). Distribution of AMF evidenced from molecular data (Davison et al., 2015) and spore morphology (Stürmer et al., 2018) reveals low levels of endemism for AMF species. There are currently 317 species of AMF morphologically described (International Culture Collection of Glomeromycota [CICG], 2020) and distributed in four orders (Diversisporales, Glomerales, Archaeosporales, and Paraglomerales), 11–16 families, and 39–49 genera depending on the classification system followed (Wijayawardene et al., 2020).

The Neotropical biogeographic realm, as delimited by the Sclater–Wallace system, includes Central America (up to central Mexico), the Caribbean Islands, and South America, and it has fascinated naturalists and biologists for its high biodiversity (Chazot et al., 2019). A revision of the Wallace biogeographical realms by Holt et al. (2013) includes the Caribbean islands and part of Central America in the Panamanian realms. The Neotropics are distributed across the North American, the Caribbean, and the South America tectonic plates (Antonelli and Sanmartín, 2011). As part of the Gondwana, the South American continent started to separate from Africa *ca.* 125 million years ago, developed its own biota during >90 million years while drifting west and relatively isolated from other continents, and became connected with North America by the Isthmus of Panama at 3.5 million years ago (Gentry, 1982; Brown and Lomolino, 2008). Diversification in the Neotropics was also affected by event of uplifting, including the formation of the Andean cordilleras which modified its climate and landscape (Hoorn et al., 2010), and by climatic fluctuations in the Pleistocene (Prance, 1973). Indeed, Neotropics includes 11 out of 14 terrestrial biomes recognized by Olson et al. (2001): Desert and Xeric Shrublands (DXS), Mangroves (MAN), Mediterranean Forests (MED), Montane Grasslands and Shrublands (MON), Temperate Broadleaf and Mixed Forests (TMF), Temperate Grasslands (TGS), Tropical and Subtropical Dry Forests (SDF), Tropical and Subtropical Grasslands (SGS), Tropical and Subtropical Moist Forests (SBF), Flooded Grasslands (FGS), and Tropical and Subtropical Coniferous Forests (SCF).

Occurrence and diversity of Glomeromycota in Neotropical biomes have been investigated, mainly in Brazil, Argentina, and Chile (Cofré et al., 2019). These studies emphasized comparison among distinct habitats (Castillo et al., 2005; Stürmer and Bellei, 1994; Carvalho et al., 2012; Pereira et al., 2014), variation along environmental gradients (Lugo et al., 2008; Silva et al., 2014; Coutinho et al., 2015; Velázquez et al., 2016a,b), and seasonal variation of AMF population (Lugo and Cabello, 2002; Escudero and Mendoza, 2005; Stürmer and Siqueira, 2011). Moreover, diversity of AMF was assessed in agricultural ecosystems (Siqueira et al., 1989; Carrenho et al., 2001; Aguilera et al., 2014), mining sites (Schneider et al., 2013; Souza et al., 2013), and habitats under revegetation (Cuenca and Lovera, 1992). Checklists of Glomeromycota species have been produced for some ecoregions within the Neotropics like the Brazilian Cerrado (Jobim et al., 2016), Caatinga (Goto et al., 2010), and Atlantic rainforest (Jobim et al., 2018). Cofré et al. (2019) compiled results of AMF species occurrence in South America based on 110 articles and recorded 186 species belonging to 9 families and 24 genera. Authors emphasized the occurrence of AMF species within the distinct ecoregions in South America and recorded Amazonia, Atlantic Forest, Caatinga, and Chaco as the regions with the highest number of species detected. For Brazil, Maia et al. (2020) recorded a total of 192 AMF species in 38 genera and 15 families, which represents 60% of the total richness described for Glomeromycota. Most of the studies and species recorded in Brazilian territory were from the Atlantic rainforest, the Cerrado savanna, and the Caatinga dry forest, emphasizing that Brazilian ecosystems are important repository for this group of soil fungi.

In this work, a systematic biogeography method (Parenti and Ebach, 2009) was used to analyze data from a Glomeromycota biogeography database (BD) that includes information of AMF distribution obtained from published literature, accession database from living culture collections, and species description (Stürmer et al., 2018). Our goal is to provide an updated checklist of AMF species and genera detected in the Neotropics and elucidate AMF distribution patterns according to the major Neotropical biomes.

## Materials and Methods

The BD is organized with four main classes of information: taxonomic (data on order, family, genus and species), geographic (data on biogeographical realm, biomes, ecoregions, continent, country, state, location, plant host, latitude and longitude, hemisphere, and climatic zone), environmental (data on soil pH, P, N, C, and organic matter, and temperature, precipitation and altitude), and origination category (indicating whether data came from species description, records from INVAM (International Culture Collection of (Vesicular) Arbuscular Mycorrhizal Fungi, Morgantown, WV, United States) and CICG (International Culture Collection of Glomeromycota, Blumenau, SC, Brazil), or other published manuscript) (Stürmer et al., 2018). Biogeographic realms are considered based on Holt et al. (2013) and biomes and ecoregions recognized according to Olson et al. (2001).

For this paper, we searched the BD for the term “Neotropics” and “Panamanian” as they cover the Neotropical realm according to Wallace, the concept being followed in this special issue. After that, we grouped the records by biomes and generated a list of families, genera, and AMF species occurring in each biome. A measure of frequency of occurrence of a species was calculated based on the number of records in the database and proportion of families among biomes investigated based on the number of species pertaining to a given family. Venn diagram was used to depict number of AMF species unique and shared among selected biomes which were most extensively surveyed for AMF. Jaccard’s index was used to determine the similarity of AMF species among biomes and calculated using PAST (Hammer et al., 2001).

Longitude and latitude data from manuscripts that surveyed AMF communities were used to obtain data of mean annual temperature and precipitation from WorldClim^1^. We then investigated the relationship of both climate data with total species richness and number of species belonging to Glomeraceae, Gigasporaceae, and Acaulosporaceae using linear models.

We found some taxonomic conundrum during our study that must be clarified. First, we followed Bentivenga and Morton (1995) who recognized only five species of *Gigaspora*. Second, species with gigasporoid mode of spore formation were considered to be part of the family Gigasporaceae as recommended by Redecker et al. (2013). Third, *Fuscutata aurea* was considered in our species list since it was not considered by Redecker et al. (2013) when species of *Fuscutata* were transferred to *Dentiscutata*. Fourth, we followed the arrangement proposed by Bills and Morton (2015) for *Ambispora*. Fifth, we followed the proposal of Walker et al. (2017) to conserve the name *Rhizophagus* but some species are referred herein as *Rhizoglomus* only because of the lack of nomenclatural combination to the genus *Rhizophagus*, since molecular phylogenetic data clearly put these species in the *Rhizophagus* clade.

## Results

**FIGURE 1:**
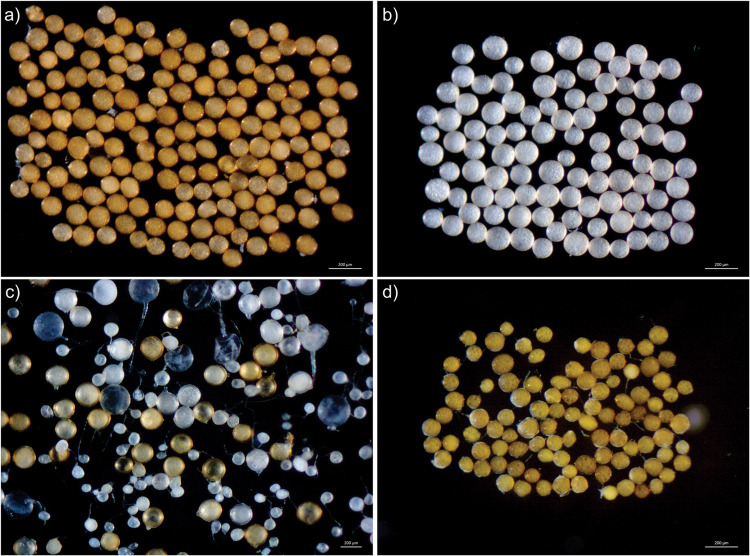
Spores of the most frequent species of Glomeromycota in the Neotropics: **(a)** *Acaulospora mellea*, **(b)** *Acaulospora scrobiculata*, **(c)** *Ambispora leptoticha*, **(d)** *Claroideoglomus etunicatum*. Source: International Culture Collection of Glomeromycota (www.furb.br/cicg).

A total of 5178 records (out of 10,961) were recovered of the biogeography database for the Neotropical realm, which originated from 178 articles published and 62 accessions of INVAM and CICG (Supplementary File 1). The number of AMF species recorded for the Neotropics is 221, which represents 69% of the 317 species (International Culture Collection of Glomeromycota [CICG], 2020) described up to date for the phylum Glomeromycota (Table 1). All orders (Archaeosporales, Paraglomerales, Diversisporales, and Glomerales) were detected and taxa pertained to 37 genera in the families Glomeraceae (76 species), Acaulosporaceae (47 species), Gigasporaceae (51 species), Diversisporaceae (15 species), Paraglomeraceae (7 species), Archaeosporaceae (5 species), Pacisporaceae (6 species), Claroideoglomeraceae (6 species), Ambisporaceae (4 species), Pervetustaceae (1 species), and Sacullosporaceae (1 species). *Entrophospora infrequens* (*Incertae sedis*) was also recorded but not assigned to any family, following the recommendation of Redecker et al. (2013). The most frequent species occurring in the Neotropics based on the number of records were *Claroideoglomus etunicatum* (185 records), *Acaulospora scrobiculata* (212 records), *Acaulospora mellea* (164 records), and *Ambispora leptoticha* (146 records) (Figure 1). *Acaulospora mellea*, *Claroideoglomus etunicatum*, and *Ambispora leptoticha* were the only species detected in ten biomes and 63 species were detected in only one biome. Seventy-six species of AMF (24% of the phylum) were described from type specimens found in the Neotropics (Supplementary Table 1). We also found six heterotypic synonym based on specimens collected from the Neotropics (Supplementary Table 1).

**TABLE 1:** Families and species of Glomeromycota occurring in Neotropics biomes.

Families/*species*	DXS	MAN	MED	MON	TMF	TGS	SDF	SGS	SBF	FGS	SCF	Records
**Paraglomeraceae**												
*Paraglomus albidum* (C. Walker & L.H. Rhodes) Oehl, F.A. Souza, G.A. Silva & Sieverd.									x			2
*Paraglomus bolivianum* (Sieverd. & Oehl) Oehl & G.A. Silva						x	x	x	x			16
*Paraglomus brasilianum* (Spain & J. Miranda) J.B. Morton & D. Redecker		x		x		x		x	x			16
*Paraglomus laccatum* (Błaszk.) C. Renker, Błaszk. & Buscot					x	x			x			8
*Paraglomus lacteum* (S.L. Rose & Trappe) Oehl, F.A. Souza, G.A. Silva & Sieverd.									x			2
*Paraglomus occidentale* Corazon-Guivin, G.A. Silva & Oehl									x			1
*Paraglomus occultum* (C. Walker) J.B. Morton & D. Redecker	x	x	x	x	x		x	x	x	x		94
*Paraglomus pernambucanum* Oehl, C.M. Mello, Magna & G.A. Silva							x		x			15
**Pervetustaceae**												
*Pervetustus simplex* Błaszk., Chwat, Kozłowska, Crossay, Symanczik & Al-Yahya’ei							x					5
**Ambisporaceae**												
*Ambispora gerdemannii* (S.L. Rose, B.A. Daniels & Trappe) R.J. Bills & J.B. Morton comb. nov.			x		x	x	x	x	x			23
*Ambispora leptoticha* (N.C. Schenck & G.S. Sm.) R.J. Bills & J.B. Morton		x	x	x	x	x	x	x	x	x	x	160
*Ambispora nicolsonii* (C. Walker, L.E. Reed & F.E. Sanders) Oehl, G.A. Silva, B.T. Goto & Sieverd.						x			x			5
*Ambispora reticulata* Oehl & Sieverd.					x				x			4
**Archaeosporaceae**												
*Archaeospora ecuadoriana* A. Schüßler & C. Walker									x			1
*Archaeospora myriocarpa* (Spain, Sieverd. & N.C. Schenck) Oehl, G.A. Silva, B.T. Goto & Sieverd.			x		x			x	x			8
*Archaeospora schenkii* (Sieverd. & S. Toro) C. Walker & Schüßler		x			x		x		x		x	15
*Archaeospora trappei* (R.N. Ames & Linderman) J.B. Morton & D. Redecker		x	x		x	x	x	x	x			54
*Archaeospora undulata* (Sieverd.) Sieverd., G.A. Silva, B.T. Goto & Oehl		x	x		x			x	x			12
**Acaulosporaceae**												
*Acaulospora alpina* Oehl, Sýkorová & Sieverd.			x		x	x		x	x			20
*Acaulospora aspera* Corazon-Guivin, Oehl & G.A. Silva									x			1
*Acaulospora baetica* Palenz., Oehl, Azcón-Aguilar & G.A. Silva									x			3
*Acaulospora bireticulata* F.M. Rothwell & Trappe					x	x	x	x	x			45
*Acaulospora brasiliensis* (B.T. Goto, L.C. Maia & Oehl) C. Walker, M. Krüger & A. Schüßler								x	x			12
*Acaulospora capsicula* Blaszk.								x	x			4
*Acaulospora cavernata* Błaszk.					x	x		x	x			16
*Acaulospora colombiana* (Spain & N.C. Schenck) Kaonongbua, J.B. Morton & Bever		x					x	x	x	x		69
*Acaulospora colossi* P.A. Schultz, Bever & J.B. Morton					x			x	x			10
*Acaulospora delicta* C. Walker, C.M. Fief. & Bliss					x	x	x	x	x			41
*Acaulospora denticulata* Sieverd. & S. Toro		x				x	x	x	x			50
*Acaulospora dilatata* J.B. Morton					x	x	x					18
*Acaulospora elegans* Trappe & Gerd.						x	x		x			11
*Acaulospora endographis* B.T. Goto									x			3
*Acaulospora entreriana* M.S. Velázquez & Cabello				x		x	x	x	x			14
*Acaulospora excavata* Ingleby & C. Walker		x				x	x	x	x			49
*Acaulospora foveata* Trappe & Janos		x			x	x	x	x	x			118
*Acaulospora gedanensis* Błaszk.							x		x			3
*Acaulospora herrerae* Furrazola, B.T.Goto, G.A.Silva, Sieverd. & Oehl							x	x	x			17
*Acaulospora ignota* Blaszk., Góralska, Chwat & Goto		x							x			2
*Acaulospora kentinensis* (C.G. Wu & Y.S. Liu) Kaonongbua, J.B. Morton & Bever		x					x	x	x		x	13
*Acaulospora koskei* Błaszk.					x			x	x			23
*Acaulospora lacunosa* J.B. Morton						x	x	x	x		x	7
*Acaulospora laevis* Gerd. & Trappe		x	x	x	x	x	x	x	x		x	106
*Acaulospora longula* Spain & N.C. Schenck		x	x	x	x		x	x	x	x		59
*Acaulospora mellea* Spain & N.C. Schenck	x	x	x		x	x	x	x	x	x	x	178
*Acaulospora minuta* Oehl, Tchabi, Hount., Palenz., I.C. Sánchez & G.A. Silva							x					5
*Acaulospora morrowiae* Spain & N.C. Schenck		x					x	x	x	x	x	135
*Acaulospora nivalis* Oehl, Palenz., I.C. Sánchez, G.A. Silva & Sieverd.								x				2
*Acaulospora papillosa* C.M.R. Pereira & Oehl							x		x			4
*Acaulospora paulineae* Błaszk.			x		x	x	x	x	x			24
*Acaulospora polonica* Błaszk.									x			2
*Acaulospora punctata* Oehl, Palenz., I.C. Sánchez, G.A. Silva, C. Castillo & Sieverd.			x		x				x			10
*Acaulospora reducta* Oehl, B.T. Goto & C.M.R. Pereira						x	x	x	x			13
*Acaulospora rehmii* Sieverd. & S. Toro		x				x	x	x	x			75
*Acaulospora rugosa* J.B. Morton				x				x	x			10
*Acaulospora scrobiculata* Trappe		x		x	x	x	x	x	x	x	x	234
*Acaulospora sieverdingii* Oehl, Sýkorová, Błaszk. & G.A. Silva			x		x		x	x	x			13
*Acaulospora spinosa* C. Walker & Trappe				x	x	x	x	x	x	x	x	129
*Acaulospora spinosissima* Oehl, Palenz., I.C. Sánchez, Tchabi, Hount. & G.A. Silva								x	x			12
*Acaulospora spinulifera* Oehl, V.M. Santos, J.S. Pontes & G.A. Silva								x				1
*Acaulospora splendida* Sieverd., Chaverri & I. Rojas							x		x			5
*Acaulospora sporocarpia* S.M. Berch									x			1
*Acaulospora thomii* Błaszk.			x		x							3
*Acaulospora tuberculata* Janos & Trappe						x	x	x	x	x		72
*Acaulospora verna* Błaszk.								x				2
*Acaulospora walkeri* Kramad. & Hedger							x	x	x			8
***Incertae saedis***												
*Entrophospora infrequens* (I.R. Hall) R.N. Ames & R.W. Schneid.	x	x			x	x	x	x	x		x	81
**Diversisporaceae**												
*Corymbiglomus corymbiforme* (Błaszk.) Błaszk. & Chwat									x			2
*Corymbiglomus globiferum* (Koske & C. Walker) Błaszk. & Chwat							x	x	x			10
*Corymbiglomus pacificum* Oehl, Medina, P. Cornejo, Sánchez-Castro, G.A. Silva & Palenz.					x							1
*Diversispora aurantia* (Błaszk., Blanke, Renker & Buscot) C. Walker & A. Schüßler									x			6
*Diversispora eburnea* (L.J. Kenn., J.C. Stutz & J.B. Morton) C. Walker & A. Schüßler		x					x	x	x			10
*Diversispora pustulata* (Koske, Fries, C. Walker & Dalpé) Oehl, G.A. Silva & Sieverd.									x			4
*Diversispora spurca* (C.M. Pfeiff., C. Walker & Bloss) C. Walker & A. Schüßler	x		x		x		x	x	x			28
*Diversispora trimurales* (Koske & Halvorson) C. Walker & A. Schüßler									x			4
*Diversispora versiformis* (P. Karst.) Oehl, G.A. Silva & Sieverd.			x		x		x		x			12
*Otospora bareae* J. Palenzuela, N. Ferrol & Oehl									x			1
*Redeckera fulva* (Berk. & Broome) C. Walker & A. Schüßler							x	x	x			5
*Redeckera megalocarpum* (D. Redecker) C. Walker & A. Schüßler									x			1
*Redeckera pulvinatum* (Henn.) C. Walker & A. Schüßler	x											1
*Sieverdingia tortuosa* (N.C. Schenck & G.S. Sm.) Błaszk., Niezgoda & B.T. Goto	x				x	x	x	x	x			38
*Tricispora nevadensis* (Palenzuela, Ferrol, Azcón-Aguilar & Oehl) Oehl, Palenzuela, G.A. Silva & Sieverd.							x					2
**Gigasporaceae**												
*Bulbospora minima* Oehl, Marinho, B.T. Goto & G.A. Silva							x	x	x			15
*Cetraspora armeniaca* (Błaszk.) Oehl, F.A. de Souza & Sieverd.									x			3
*Cetraspora auronigra* Oehl, L.L. Lima, Kozovits, Magna & G.A. Silva									x			3
*Cetraspora gilmorei* (Trappe & Gerd.) Oehl, F.A. de Souza & Sieverd.	x	x	x	x	x	x	x	x	x			44
*Cetraspora nodosa* (Blaszk.) Oehl, G.A. Silva, B.T. Goto & Sieverd.		x		x			x		x			18
*Cetraspora pellucida* (T.H. Nicolson & N.C. Schenck) Oehl, F.A. de Souza & Sieverd.		x				x	x	x	x	x	x	104
*Dentiscutata biornata* (Spain, Sieverd. & S. Toro) Sieverd., F.A. de Souza & Oehl						x	x	x	x	x		62
*Dentiscutata cerradensis* (Spain & J. Miranda) Sieverd., F.A. de Souza & Oehl							x	x	x			48
*Dentiscutata colliculosa* B.T. Goto & Oehl							x		x			6
*Dentiscutata erythropus* (Koske & C. Walker) C. Walker & D. Redecker							x	x	x		x	24
*Dentiscutata hawaiiensis* (Koske & Gemma) Sieverd., F.A. Souza & Oehl									x			2
*Dentiscutata heterogama* (T.H. Nicolson & Gerd.) Sieverd., F.A. de Souza & Oehl		x			x	x	x	x	x	x		125
*Dentiscutata nigra* (J.F. Readhead) Sieverd., F.A. de Souza & Oehl							x	x	x			6
*Dentiscutata reticulata* (Koske, D.D. Miller & C. Walker) Sieverd., F.A. de Souza & Oehl								x	x			9
*Dentiscutata savannicola* (R.A. Herrera & Ferrer) Walker, Krüger & Schüßler				x			x	x	x			19
*Dentiscutata scutata* (C. Walker & Dieder.) Sieverd., F.A. de Souza & Oehl							x	x	x		x	33
*Fuscutata aurea* Oehl, C.M. Mello & G.A. Silva								x	x			4
*Gigaspora albida* N.C. Schenck & G.S. Sm.	x	x	x				x	x	x			46
*Gigaspora decipiens* I.R. Hall & L.K. Abbott		x				x	x	x	x			98
*Gigaspora gigantea* (T.H. Nicholson & Gerd.) Gerd. & Trappe		x				x	x	x	x		x	84
*Gigaspora margarita* W.N. Becker & I.R. Hall		x				x	x	x	x	x	x	136
*Gigaspora rosea* T.H. Nicolson & N.C. Schenck						x	x	x	x			30
*Intraornatospora intraornata* (B.T. Goto & Oehl) B.T. Goto, Oehl & G.A. Silva							x		x			17
*Paradentiscutata bahiana* Oehl, Magna, B.T. Goto & G.A. Silva							x		x			9
*Paradentiscutata maritima* B.T. Goto, D.K. Silva, Oehl & G.A. Silva		x					x		x			11
*Racocetra alborosea* (Ferrer & R.A. Herrera) Oehl, F.A. de Souza & Sieverd.								x	x		x	7
*Racocetra castanea* (C. Walker) Oehl, F.A. de Souza & Sieverd.							x	x	x			13
*Racocetra coralloidea* (Trappe, Gerd. & I. Ho) Oehl, F.A. de Souza & Sieverd.		x				x	x	x	x			49
*Racocetra crispa* F.A. de Souza, I. R. Silva, M.B. Barros-Barreto, B.T. Goto & Oehl								x				1
*Racocetra fulgida* (Koske & C. Walker) Oehl, F.A. de Souza & Sieverd.		x				x	x	x	x		x	53
*Racocetra gregaria* (N.C. Schenck & T.H. Nicolson) Oehl, F.A. de Souza & Sieverd.							x	x	x	x		31
*Racocetra minuta* (Ferrer & R.A. Herrera) Oehl, F.A. de Souza & Sieverd.		x						x	x			7
*Racocetra persica* (Koske & C. Walker) Oehl, F.A. de Souza & Sieverd.							x	x	x			16
*Racocetra tropicana* Oehl, B.T. Goto & G.A. Silva		x					x	x	x			20
*Racocetra verrucosa* (Koske & C. Walker) Oehl, F.A. de Souza & Sieverd.							x	x	x			21
*Scutellospora alterata* Oehl, J.S. Pontes, Palenz., Sánchez-Castro & G.A. Silva							x					2
*Scutellospora arenicola* Koske & Halvorson									x			3
*Scutellospora aurigloba* (I.R. Hall) C.Walker & F.E. Sanders					x		x		x		x	17
*Scutellospora calospora* (T.H. Nicolson & Gerd.) C. Walker & F.E. Sanders		x	x		x	x	x	x	x		x	90
*Scutellospora crenulata* R.A. Herrera-Peraza, Cuenca & C. Walker								x				1
*Scutellospora dipapillosa* (C. Walker & Koske) C. Walker & F.E. Sanders						x		x			x	12
*Scutellospora dipurpurescens* J.B. Morton & Koske			x		x		x		x			23
*Scutellospora pernambucana* Oehl, D.K Silva, N. Freitas, L.C. Maia		x					x	x	x			36
*Scutellospora projecturata* Kramad. & C. Walker		x					x		x			4
*Scutellospora rubra* Stürmer & J.B. Morton						x	x	x	x	x		31
*Scutellospora spinosissima* C. Walker & Cuenca				x			x	x	x			22
*Scutellospora striata* Cuenca & Herrera								x	x			2
*Scutellospora tepuiensis* Furrazola & Cuenca								x				1
*Scutellospora tricalypta* (R.A. Herrera & Ferrer) C. Walker & F.E. Sanders									x			1
*Scutellospora weresubiae* Koske & C. Walker		x			x		x	x	x			21
**Pacisporaceae**												
*Pacispora chimonobambusae* (C.G. Wu & Y.S. Liu) Sieverd. & Oehl ex C Walker, Vestberg & Schüßler									x			1
*Pacispora dominikii* (Blaszk.) Sieverd. & Oehl			x		x			x	x			9
*Pacispora franciscana* Oehl & Sieverd.		x					x					4
*Pacispora patagonica* (Novas & Fracchia) C. Walker, Vestberg & Schüßler					x	x		x				6
*Pacispora robigina* Oehl & Sieverd.								x	x			6
*Pacispora scintillans* (S. L. Rose & Trappe) Sieverd. & Oehl ex C. Walker, Vestberg & Schüßler						x	x					4
**Sacculosporaceae**												
*Sacculospora baltica* (Błaszk., Madej & Tadych) Oehl, Palenz., Sánchez-Castro, B.T. Goto, G.A. Silva & Sieverd.									x			3
**Claroideoglomeraceae**												
*Claroideoglomus claroideum* (N.C. Schenck & G.S. Sm.) C. Walker & A. Schüßler		x	x	x	x	x	x	x	x		x	105
*Claroideoglomus drummondii* (Blaszk. & C. Renker) C. Walker & A. Schüßler									x			7
*Claroideoglomus etunicatum* (W.N. Becker & Gerd.) C. Walker & A. Schüßler	x	x	x	x	x	x	x	x	x	x	x	204
*Claroideoglomus lamellosum* (Dalpé, Koske & Tews) C. Walker & A. Schüßler			x					x	x			16
*Claroideoglomus luteum* (L.J. Kenn., J.C. Stutz & J.B. Morton) C. Walker & A. Schüßler						x	x	x	x		x	25
*Claroideoglomus walker* (Blaszk. & C. Renker) C. Walker & A. Schüßler									x			3
**Glomeraceae**												
*Dominikia aurea* (Oehl & Sieverd.) Blaszk. Chwat, G.A. Silva & Oehl			x				x		x			8
*Dominikia bernensis* Oehl, Palenz., Sánchez-castro & G.A. Silva							x					1
*Dominikia indica* (Błaszk., Wubet & Harikumar) Błaszk., G.A. Silva & Oehl		x										1
*Dominikia iranica* (Błaszk., Kovács & Balázs) Błaszk., Chwat & Kovács									x			2
*Dominikia minuta* (Błaszk., Tadych & Madej) Błaszk., Chwat & Kovác	x							x	x			4
*Funneliformis badium* (Oehl, Redecker & Sieverd.) C. Walker & A. Schüßler			x		x			x				11
*Funneliformis caesaris* (Sieverd. & Oehl) Oehl, G.A. Silva & Sieverd.										x		1
*Funneliformis caledonium* (Nicolson & Gerdemann) C. Walker & A. Schüßler		x		x			x		x			23
*Funneliformis coronatum* (Giovann.) C. Walker & A. Schüßler			x	x	x	x	x		x			27
*Funneliformis dimorphicus* (Boyetchko & J.P. Tewari) Oehl, G.A. Silva & Sieverd.						x						4
*Funneliformis fragilistratum* (Skou & I. Jakobsen) C. Walker & A. Schüßler		x										1
*Funneliformis geosporum* (T.H. Nicolson & Gerd.) C. Walker & A. Schüßler	x	x	x		x	x	x	x	x		x	102
*Funneliformis halonatum* (S.L. Rose & Trappe) Oehl, G.A. Silva & Sieverd.		x					x	x	x			17
*Funneliformis kerguelensis* (Dalpé & Strullu) Oehl, G.A. Silva & Sieverd.									x			3
*Funneliformis monosporus* (Gerd. & Trappe) Oehl, G.A. Silva & Sieverd.			x				x		x			7
*Funneliformis mosseae* (T.H. Nicolson & Gerd.) C. Walker & A. Schüßler	x		x	x	x	x	x	x	x		x	130
*Funneliformis multiforum* (Tadych & Blaszk.) Oehl, G.A. Silva & Sieverd.									x			1
*Funneliformis verruculosum* (Blaszk.) C. Walker & A. Schüßler		x					x		x		x	11
*Funneliglomus sanmartinensis* Corazon-Guivin, G.A. Silva & Oehl									x			1
*Glomus aggregatum* N.C. Schenck & G.S. Sm. emend. Koske	x		x			x	x	x	x			42
*Glomus ambisporum* G.S. Sm. & N.C. Schenck		x	x			x	x	x	x			45
*Glomus australe* (Berk.) S.M. Berch									x			2
*Glomus botryoides* F.M. Rothwell & Victor									x			2
*Glomus brohultii* Sieverd. & Herrera		x			x	x	x	x	x			66
*Glomus crenatum* Furrazola, R.L. Ferrer, R.A. Herrera & B.T. Goto							x					2
*Glomus cubense* Y. Rodr. & Dalpé							x					1
*Glomus flavisporum* (M. Lange & E.M. Lund) Trappe & Gerd.									x			3
*Glomus formosanum* C.G. Wu & Z.C. Chen								x	x			3
*Glomus fuegianum* (Speg.) Trappe & Gerd.					x	x		x	x			16
*Glomus glomerulatum* Sieverd.		x				x	x	x	x		x	86
*Glomus heterosporum* G.S. Sm. & N.C. Schenck								x	x			4
*Glomus herrerae* Torres-Arias, Furrazola & B.T. Goto		x										1
*Glomus hoi* S.M. Berch & Trappe			x		x				x		x	10
*Glomus invermaium* I.R. Hall			x	x	x		x	x	x			36
*Glomus macrocarpum* Tul. & C. Tul.		x	x		x		x	x	x		x	139
*Glomus magnicaule* I.R. Hall								x	x			5
*Glomus melanosporum* Gerd. & Trappe							x				x	2
*Glomus microaggregatum* Koske, Gemma & P.D. Olexia		x			x	x	x	x	x			52
*Glomus microcarpum* Tul. & C. Tul.	x	x					x	x	x			65
*Glomus multicaule* Gerd. & B.K. Bakshi							x					9
*Glomus nanolumen* Koske & Gemma								x	x			6
*Glomus pallidum* I.R. Hall			x		x		x		x			7
*Glomus radiatum* (Thaxt.) Trappe & Gerd.											x	1
*Glomus reticulatum* Bhattacharjee & Mukerji									x			2
*Glomus segmentatum* Trappe, Spooner & Ivory									x		x	2
*Glomus spinuliferum* Sieverd. & Oeh							x	x	x		x	16
*Glomus tenebrosum* (Thaxt.) S.M. Berch							x	x	x			6
*Glomus trufemii* B.T. Goto, G. A. Silva & Oehl							x		x			15
*Halonatospora pansihalos* (S.M. Berch & Koske) Blask., Niezgoda, B.T. Goto & Kozlowska									x			1
*Microkamienskia peruviana* Corazon-Guivin, G.A. Silva & Oehl									x			3
*Nanoglomus plukenetiae* Corazon-Guivin, G.A. Silva & Oehl									x			1
*Oehlia diaphana* (J.B. Morton & C. Walker) Błaszk., Kozłowska, Niezgoda, B.T. Goto & Dalpé			x	x	x	x	x	x	x	x		49
*Rhizoglomus arabicus* Blaszk., Symanczik & Al-Yahya’ei		x					x					2
*Rhizoglomus maiae* Błaszk., Piątek, Yorou, Zubek, Jobim, Niezgoda & B.T. Goto									x			1
*Rhizoglomus variabile* Corazon-Guivin, Oehl & G.A. Silva									x			1
*Rhizophagus clarus* (T.H. Nicolson & N.C. Schenck) C. Walker & A. Schüßler		x			x	x	x	x	x	x	x	128
*Rhizophagus custos* (C. Cano & Y. Dalpé) C. Walker & A. Schüßler							x					1
*Rhizophagus fasciculatus* (Thaxt.) C. Walker & A. Schüßler		x	x	x	x	x	x	x	x			72
*Rhizophagus intraradices* (N.C. Schenck & G.S. Sm.) C. Walker & A. Schüßler	x	x	x	x	x	x	x	x	x	x	x	99
*Rhizophagus irregularis* (Błaszk., Wubet, Renker & Buscot) C. Walker & A. Schüßler				x			x	x	x			19
*Rhizophagus manihotis* (R.H. Howeler, Sieverd. & N.C. Schenck) C. Walker & A. Schüßler	x	x					x	x				5
*Rhizophagus natalensis* Błaszk., Chwat & B.T. Goto								x	x			6
*Rhizophagus proliferus* (Dalpé & Declerck) C. Walker & A. Schüßler		x		x					x			4
*Rhizophagus vesiculiferus* (Thaxt.) C. Walker & A. Schüßler		x		x	x				x			7
*Sclerocarpum amazonicum* Jobim, Błaszk., Niezgoda, Kozłowska & B.T. Goto									x			1
*Sclerocystis clavispora* Trappe					x		x	x	x			31
*Sclerocystis coremioides* Berk. & Broome							x	x	x			37
*Sclerocystis liquidambaris* C.G. Wu & Z.C. Chen									x			3
*Sclerocystis rubiformis* Gerd. & Trappe	x	x			x		x	x	x		x	29
*Sclerocystis sinuosa* Gerd. & B.K. Bakshi		x		x		x	x	x	x		x	72
*Sclerocystis taiwanensis* C.G. Wu & Z.C. Chen							x		x			19
*Septoglomus constrictum* (Trappe) Sieverd., G.A. Silva & Oehl	x	x	x	x		x	x	x	x			68
*Septoglomus deserticola* (Trappe, Bloss & J.A. Menge) G.A. Silva, Oehl & Sieverd.	x					x	x	x	x		x	10
*Septoglomus furcatum* Błaszk., Chwat & Kovács, Ryszka							x					1
*Septoglomus titan* B.T. Goto & G.A. Silva							x					2
*Septoglomus viscosum* (T.H. Nicolson) C. Walker, D. Redecker, D. Stille & A. Schüßler								x	x			9
*Septoglomus xanthium* (Błaszk., Blanke, Renker & Buscot) G.A. Silva, Oehl & Sieverd.									x			2
**Total number of species**	**19**	**63**	**40**	**27**	**59**	**63**	**127**	**124**	**186**	**20**	**38**	

*DXS, Deserts & Xeric Shrublands; MAN, Mangroves; MED, Mediterranean Forests; Woodlands & Scrub; MON, Montane Grasslands & Shrublands; TMF, Temperate Broadleaf & Mixed Forests; TGS, Temperate Grasslands, Savannas & Shrublands; SDF, Tropical & Subtropical Dry Broadleaf Forests; SGS, Tropical and Subtropical Grassslands; Savannas & Shrublands; SBF, Tropical & Subtropical Moist Broadleaf Forests; FGS, Flooded Grasslands & Savannas; SCF, Tropical and Subtropical Coniferous Forests.*

Studies were conducted in most countries of South America but there are no records in the BD from Uruguay, Paraguay, Guiana, Suriname, French Guiana, and El Salvador. The Caribbean islands are represented in the BD by Cuba, Jamaica, Guadeloupe, and Martinique. The highest numbers of records in the BD were from Brazil, Argentina, Mexico, and Colombia, from where 182, 77, 87, and 77 species were recorded, respectively (Supplementary Table 2).

**FIGURE 2:**
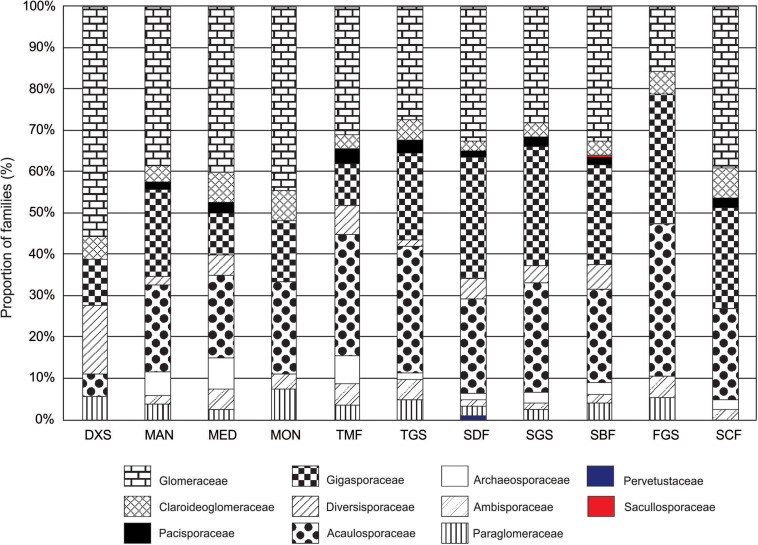
Proportion of families (%) in Glomeromycota based on the number of species per family in Neotropical biomes. DXS, Desert and Xeric Shrublands; MAN, Mangroves; MED, Mediterranean Forests, Woodlands & Scrub; MON, Montane Grasslands & Shrublands; TMF, Temperate Broadleaf & Mixed Forests; TGS, Temperate Grasslands; Savannas & Shrublands; SDF, Tropical & Subtropical Dry Broadleaf Forests; SGS, Tropical and Subtropical Grasslands; Savannas & Shrublands; SBF, Tropical & Subtropical Moist Broadleaf Forests. FGS, Flooded Grasslands & Savannas; SCF, Tropical and Subtropical Coniferous Forests.

AMF species were registered from all 11 biomes occurring in the Neotropical realm, and distribution of families based on the number of species differed among biomes (Figure 2). Six families were detected for DXS and MON, and seven families for SCF, while nine to ten families were reported for other biomes. Pervetustaceae and Sacullosporaceae were recorded only for SDF and SBF, respectively, while Archaeosporaceae, Diversisporaceae, and Pacisporaceae were not detected for FGS and MON. Paraglomeraceae and Archaeosporaceae were not recorded for SCF and DXS, respectively. Species in FGS pertained mainly to Acaulosporaceae and Gigasporaceae which together accounted for 68.4% of the total number of species. Glomeraceae accounted for 32.6% to 55.6% of species in DXS, MAN, MED, MON, SBF, and SCF, while for TMF, TGS, SDF, and SGS families Glomeraceae, Acaulosporaceae, and Gigasporaceae were more evenly distributed.

**FIGURE 3:**
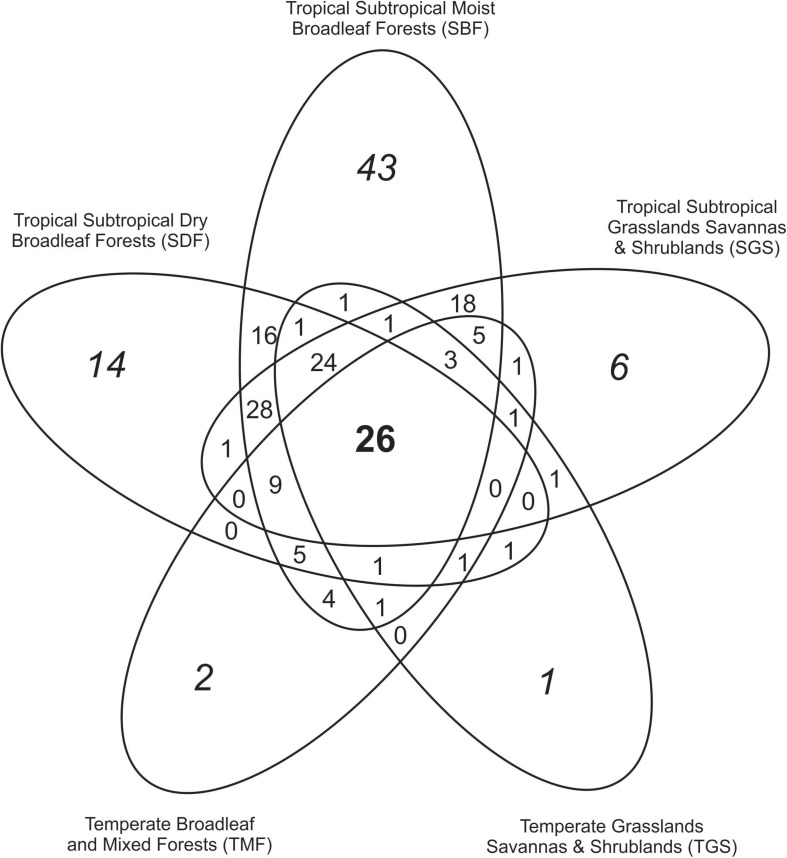
Venn diagram showing the number of species in Glomeromycota that are unique and shared among selected Neotropical biomes.

Biomes TMF, TGS, SDF, SGS, and SBF harbored the largest number of species and 26 species were shared among them (Figure 3). These species pertained to 13 genera distributed in the families Ambisporaceae, Archaeosporaceae, Acaulosporaceae, Gigasporaceae, Diversisporaceae, Claroideoglomeraceae, and Glomeraceae. Species shared by two, three, and four biomes ranged mostly from 0–9; however, this number ranged from 18 to 28 when SGS and SBF were included in the comparison (Figure 3). The highest number of exclusive species was detected in SBF (43 species) followed by SDF (14 species) and SGS (6 species). Species were detected in 52 different ecoregions within biomes in the Neotropics (Supplementary Table 3). Ecoregions Caatinga, Cerrado, and Serra do Mar coastal forest harbored 98, 92, and 86 species, respectively, and were the ecoregions with the highest number of species recorded (Supplementary Table 3).

We investigated patterns of similarity of AMF species composition among Neotropical biomes using Jaccard’s index (Table 2). Similarity among most biomes ranged from 9% to 43% (Table 2), and the average similarity among all biomes was 25%. The largest number of AMF species was recorded for SBF (186 species), SDF (127 species), and SGS (124 species) (Table 1), and similarity among these biomes ranged from 53 to 57%. Similarity of MED and MON with other biomes ranged from 13 to 26%, except MED and TMF that shared 50% of species.

**TABLE 2:** Jaccard’s similarity of arbuscular mycorrhizal fungi species between biomes of the Neotropical biogeography realm.

	DXS	MAN	MED	MON	TMF	TGS	SDF	SGS	SBF	FGS
MAN	0.18									
MED	0.23	0.22								
MON	0.15	0.25	0.26							
TMF	0.16	0.27	0.50	0.23						
TGS	0.15	0.30	0.24	0.22	0.37					
SDF	0.13	0.33	0.22	0.18	0.28	0.40				
SGS	0.14	0.30	0.23	0.17	0.33	0.43	0.55			
SBF	0.09	0.26	0.20	0.15	0.28	0.31	0.53	0.57		
FGS	0.12	0.20	0.13	0.21	0.16	0.21	0.15	0.15	0.10	
SCF	0.15	0.30	0.15	0.15	0.23	0.33	0.27	0.23	0.20	0.20

*See Figure 2 for abbreviation of biomes.*

**FIGURE 4:**
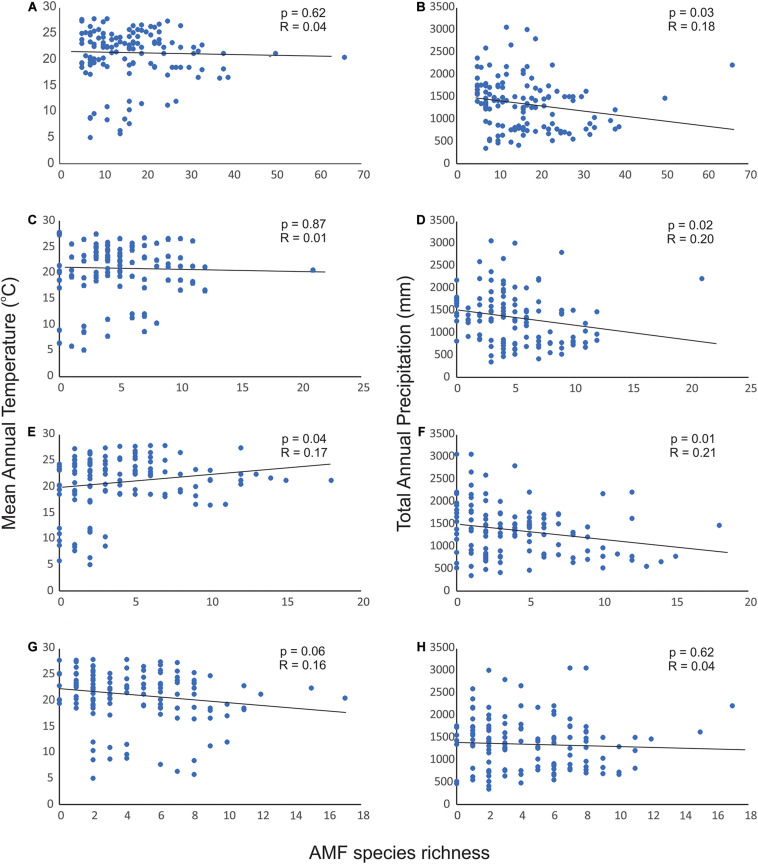
Relationship between mean annual temperature and total precipitation with total AMF species richness **(A,B)**, richness of Glomeraceae **(C,D)**, richness of Gigasporaceae **(E,F)**, and richness of Acaulosporaceae **(G,H)** in the Neotropics.

We found a very weak negative relationship between total number of AMF species and mean annual temperature and total precipitation; however, this relation was significant only for total precipitation (Figure 4). The linear models indicated that number of species in Gigasporaceae had a significant positive relationship with mean annual temperature and a significant negative relationship with total precipitation while number of species in Acaulosporaceae showed no significant relationship with both climatic data (Figure 4).

## Discussion

This study improves our knowledge on AMF distribution in the Neotropics following the contribution of Cofré et al. (2019) for South America and Maia et al. (2020) for Brazil. While Cofré et al. (2019) focused on reporting AMF species richness in distinct ecoregions in South America and Maia et al. (2020) focused on Brazilian floristic domains, our study converged to reveal distribution patterns among biomes occurring in the Neotropics. We found that 221 species of AMF have been registered from Neotropics, representing 69% of the total diversity of the phylum Glomeromycota. This result indicates that the Neotropical realm shelters a significant portion of the total diversity of the phylum, despite that some biomes and ecoregions are poorly surveyed for AMF species. For instance, most of the surveys carried in tropical forests in the Neotropics are from ecoregions pertaining to the Atlantic forest in the eastern region of Brazil while few studies were done in ecoregions of the Amazon basin. Desert and Xeric Shrublands, Flooded Grasslands, Mangroves, Mediterranean Forests, Montane Grasslands and Shrublands, and Tropical and Subtropical Coniferous Forests have very few studies on AMF occurrence compared to other biomes. Survey types of studies for AMF with a strong taxonomic basis should be encouraged in the Neotropics considering that some biodiversity hotspots are found in this realm (Myers et al., 2000) and new taxa have been discovered in the Neotropics in the last decade (*e.g.*, Goto et al., 2012; Blaszkowski et al., 2013; Jobim et al., 2019). Moreover, investigation of the AMF community associated with native and economically important plants occurring in the Neotropics like *Ilex paraguariensis* and *Araucaria angustifolia* (Moreira et al., 2007) might reveal new and unreported AMF species.

All families and genera in Glomeromycota were represented in the Neotropics, even families like Sacculosporaceae and Pervetustaceae which are populated by two and one species, respectively, and described from Europe and Asia were recorded for this realm. Glomeraceae, Acaulosporaceae, and Gigasporaceae could be considered the most representative families in the Neotropics considering the absolute number of species registered within each family. However, these families are the most speciose families within the phylum with 57–123 species described followed by Diversisporaceae (34 species), while other families are formed by 1–9 species. Considering the widespread distribution of AMF species in global ecosystems (Davison et al., 2015; Stürmer et al., 2018), it is not surprising that these three families are the most representative in the Neotropics. We suggest that representativeness of supraspecific taxa should be investigated based on the proportion of species relative to the total number of species described for that taxa. For instance, when this approach is used, Gigasporaceae, Ambisporaceae, and Acaulosporaceae are the most representative families as 89%, 80%, and 79% of the species within each family have been reported in the Neotropics. A drawback of this approach is for monospecific families and genera as their representativeness will be always 100% once the species has been registered.

Among the four most frequent species in the Neotropics detected herein, only *Ambispora leptoticha* was not included in the list of frequent species in South America compiled by Cofré et al. (2019). The possible reason for this is that these authors considered *Ambispora appendicula*, *A. callosa*, *A. fecundispora*, and *A. jimgerdemannii* as distinct species while we considered them to be synonymous with *A. leptoticha* (Bills and Morton, 2015). The four most frequent species detected in the Neotropics are not exclusive of this biogeographic realm as they were recorded in other five continents and considered to be cosmopolitan (Stürmer et al., 2018). Conversely, it is interesting that 29% of the species detected herein were found in only one biome within the Neotropics, which might be an indicative that they are rare species or associated with a particular soil or climate condition. Interesting is that the most frequent species belongs to three genera (*Ambispora*, *Acaulospora*, and *Claroideoglomus*), from three distinct families and orders. This suggests that AMF communities are composed by co-occurring fungi with different life-history strategies (Hart and Reader, 2002).

Most of AMF species in the Neotropics were recorded in five biomes (TMF, TGS, SDF, SGS, and SBF) which had the largest number of records in the BD, showing clearly a bias of studies toward these biomes. Mangroves, Flooded Grasslands, and Desert and Xeric Shrublands are biomes underrepresented in the BD, and further studies in these biomes will certainly contribute to our knowledge on AMF distribution in the Neotropics. Only a small set (26 species) of the total number of species in the Neotropics were shared among these five biomes, and they pertained to families that are basal and derived within the Glomeromycota phylogeny. This possibly indicates a characteristic of AMF assemblages: they are composed by species pertaining to basal and derived clades of Glomeromycota, regardless of the geographical location and environmental condition. However, this hypothesis has to be further tested more rigorously. Higher similarity among all Neotropical biomes was detected among SBF, SGS, and SDF, all of them being located above the 35° of latitude South, bordering each other, and with the larger number of studies and species recorded. On average, similarity among biomes within the Neotropics was 25% which is lower than the average value of similarity among all terrestrial biomes (43 to 56%) calculated by Davison et al. (2015); Stürmer et al. (2018). A possible reason for this lower similarity among biomes within the Neotropics lies on the Jaccard’s index which considers the total number of species detected in one biome. Considering that some biomes were less surveyed for AMF (*e.g.*, MON, MED), their lower number of species decreases the similarity when compared with other biomes with higher number of species.

The relationship between AMF diversity and richness to precipitation and temperature is important considering the global change scenarios (Van der Heijden et al., 2008) and the influence of AMF diversity on plant community diversity and productivity (Van der Heijden et al., 1998). Under field conditions, precipitation had a weak direct effect upon AMF richness but it influenced plant cover which in turn affected AMF species richness (Zhang et al., 2016). Experimental trials showed the increased precipitation, but not warming, decreased AMF OTU richness by 16% on average compared to control treatments (Gao et al., 2016). Manipulation of rainfall regime for 2 years affected the composition of AMF community but not species richness (Deveautour et al., 2018), although responses of AMF community might take longer time to be observed (Deveautour et al., 2020). These studies were developed mostly in arid and semi-arid grasslands at the regional level, and our results at the scale of a biogeographic realm corroborates that both precipitation and mean annual temperature have a weak influence on AMF species richness in natural communities in the Neotropics. Both climatic data also did not influence species richness of the main families within Glomeromycota, although a significant but weak relationship was found between climatic data and species richness in Glomeraceae and Gigasporaceae. One reason for this lack of relationship might be the narrow range of precipitation and temperature occurring in the Neotropics (*e.g*., 68% of temperature values range between 20 and 28°C). Our results also suggest that other variables are more important in the Neotropics to determine AMF richness, such as abiotic factors like soil P and pH, and biotic factors like the plant community. Considering that AMF communities are highly unpredictable (Powell and Bennett, 2016), species richness might be more influenced by random process or fungal dispersal capacity rather than climatic processes.

In conclusion, the Glomeromycota is well represented in Neotropical biomes and ecoregions and this biogeographic realm is a source for new species and supraspecific taxa which certainly contributes to our knowledge of the fungal dimension of biodiversity. Considering that anthropogenic actions are a threat to biodiversity in the Neotropics, assessment of AMF species composition in understudied ecoregions and biomes is urgent not only to detect new species and occurrences but also to establish these fungi in single-species culture for further use. In this point, we agree with Cofré et al. (2019) that it is important to generate species inventories from Neotropics ecosystems and obtain and deposit single culture in culture collections. Some patterns detected herein among biomes might be biased due to the few studies that have been conducted in some biomes and ecoregions. Indeed, most studies and species have been reported from Tropical and Subtropical Forests, Tropical and Subtropical Grasslands, and Tropical Dry Forests. Some biodiversity hotspots recognized by Myers et al. (2000) like Central Chile, Tropical Andes, and Western Ecuador are scarcely surveyed for AMF communities and efforts should be made to inventory Glomeromycota in these regions. Not only might results from these understudied hotspots reveal new species to science but also it provides information to subsidies public policies for conservation. Assessment of Glomeromycota biodiversity in distinct ecoregions of the Neotropics should be encouraged considering that these fungi play important roles that help to maintain terrestrial ecosystems.

## Data Availability Statement

The raw data supporting the conclusions of this article will be made available by the authors, without undue reservation.

## Author Contributions

SS managed the database, compiled Excel spreadsheets, and wrote the manuscript. KK analyzed the data, compiled species list and authority, and edited the manuscript. Both authors approved the final version of the manuscript.

## Conflict of Interest

The authors declare that the research was conducted in the absence of any commercial or financial relationships that could be construed as a potential conflict of interest.

## Footnotes

1 https://www.worldclim.org/data/bioclim.html

## References

[B1] Aguilera P. Cornejo P. Borie F. Barea J. M. von Baer E. Oehl F. (2014). Diversity of arbuscular mycorrhizal fungi associated with *Triticum aestivum* L. plants growing in an Andosol with high aluminum level. *Agric. Ecosyst. Environ.* 186 178–184. 10.1016/j.agee.2014.01.029

[B2] Antonelli A. Sanmartín I. (2011). Why are there so many plant species in the Neotropics? *Taxon* 60 403–414. 10.1002/tax.602010

[B3] Bentivenga S. P. Morton J. B. (1995). A monograph of the genus *Gigaspora*, incorporating developmental petterns of morphological characters. *Mycologia* 87 719–731. 10.2307/3760818

[B4] Bills R. J. Morton J. B. (2015). A combination of morphology dn 28S rRNA gene sequences provide grouping and ranking criteria to merge eight intro three *Ambispora* species (*Ambisporaceae*, *Glomeromycota*). *Mycorrhiza* 25 485–498. 10.1007/s00572-015-0626-7 25638691

[B5] Blaszkowski J. Chwat G. Kovács G. M. Gáspár B. K. Ryszka P. Orlowska E. (2013). Septoglomus fuscum and *S. furcatum*, two new species of arbuscular mycorrhizal fungi (*Glomeromycota*). *Mycologia* 105 670–680. 10.3852/12-127 23233507

[B6] Brown J. H. Lomolino M. V. (2008). *Biogeography.* Sunderland: Sinauer Associates Inc.

[B7] Brundrett M. C. Tedersoo L. (2018). Evolutionary history of mycorrhizal symbioses and global host plant diversity. *New Phytol.* 220 1108–1115. 10.1111/nph.14976 29355963

[B8] Carrenho R. Silva E. S. Trufem S. F. B. Bononi V. L. R. (2001). Successive cultivation of maize and agricultural practices on root colonization, number of spores and species of arbuscular mycorrhizal Fungi. *Braz. J. Microbiol.* 32 262–270.

[B9] Carvalho F. De Souza F. A. Carrenho R. Moreira F. M. S. Jesus E. C. Fernandes G. W. (2012). The mosaic of habitats in the high-altitude Brazilian rupestrian fields is a hotspot for arbuscular mycorrhizal Fungi. *Appl. Soil. Ecol.* 52 9–19. 10.1016/j.apsoil.2011.10.001

[B10] Castillo C. Borie F. Godoy R. Rubio R. Sieverding E. (2005). Diversity of mycorrhizal plant species and arbuscular mycorrhizal fungi in evergreen forest, deciduous forest and grassland ecosystems of Southern Chile. *J. Appl. Bot. Food Qual.* 80 40–47.

[B11] Chazot N. Willmott K. R. Lamas G. Freitas A. V. L. Piron-Prunier F. Arias C. F. (2019). Renewed diversification following miocene landscape turnover in a neotropical butterfly radiation. *Global Ecol. Biogeogr.* 28 1118–1132. 10.1111/geb.12919

[B12] Cofré M. N. Soteras F. Iglesias M. R. Velázquez S. Abarca C. Risio L. (2019). “Biodiversity of arbuscular mycorrhizal fungi in south america: a review,” in *Mycorrhizal Fungi in South America*, eds Pagano M. C. Lugo M. A. (Berlin: Springer), 49–72. 10.1007/978-3-030-15228-4_3

[B13] Coutinho E. S. Fernandes G. W. Berbara R. L. L. Valeìrio H. M. Goto B. T. (2015). Variation of arbuscular mycorrhizal fungal communities along an altitudinal gradient in rupestrian grasslands in Brazil. *Mycorrhiza* 25 627–638. 10.1007/s00572-015-0636-5 25771864

[B14] Cuenca G. Lovera M. (1992). Vesicular-arbuscular mycorrhizae in disturbed and revegetated sites from La Gran Sabana. *Venezuela. Can. J. Bot.* 70 73–79. 10.1139/b92-009

[B15] Davison J. Moora M. Öpik M. Adholeya A. Ainsaar L. Bâ A. (2015). Global assessment of arbuscular mycorrhizal fun- gus diversity reveals very low endemism. *Science* 349 970–973. 10.1126/science.aab1161 26315436

[B16] Deveautour C. Donn S. Power S. A. Bennett A. E. Powell J. R. (2018). Experimentally altered rainfall regimes and host root traits affect grasslands arbuscular mycorrhizal fungal communities. *Mol. Eco.* 27 2152–2163. 10.1111/mec.14536 29443420

[B17] Deveautour C. Power S. A. Barnett K. L. Ochoa-Hueso R. Donn S. Bennett A. E. (2020). Temporal dynamics of mycorrhizal fungal communities and co-associations with grassland plant communities following experimental manipulation of rainfall. *J. Ecol. Soc.* 108 515–527. 10.1111/1365-2745.13267

[B18] Dotzler N. Krings M. Taylor T. N. Agerer R. (2006). Germination shields in *Scutellospora* (*Glomeromycota*: *Diversisporales*, *Gigasporaceae*) from the 400 million-year-old Rhynie chert. *Mycol. Prog.* 5 178–184. 10.1007/s11557-006-0511-z

[B19] Dotzler N. Walker C. Krings M. Hass H. Kerp H. Taylor T. N. (2009). Acaulosporoid glomeromycotan spores with a germination shield from the 400-million-year-old Rhynie chert. *Mycol. Prog.* 8 9–18. 10.1007/s11557-008-0573-1

[B20] Escudero V. Mendoza R. (2005). Seasonal variation of arbuscular mycorrhizal fungi in temperature grassland along a wide hydrologic gradient. *Mycorrhiza* 15 291–299. 10.1007/s00572-004-0332-3 15517421

[B21] Gao C. Kim Y.-C. Zheng Y. Yang W. Chen L. Ji N.-N. (2016). Increased precipitation, rather than warming, exerts a strong influence on arbuscular mycorrhizal fungal community in a semiarid steppe ecosystem. *Botany* 94 459–469. 10.1139/cjb-2015-0210

[B22] Gentry A. H. (1982). Neotropical floristic diversity: phytogeographical connections between Central and South America, Pleistocene climatic fluctuations, or an accident of the andean orogeny? *Ann. Miss. Bot. Garden.* 69 557–593. 10.2307/2399084

[B23] Goto B. T. da Silva G. A. Yano-Melo A. M. Maia L. C. (2010). Checklist of the arbuscular mycorrhizal fungi (*Glomeromycota*) in the Brazilian semiarid. *Mycotaxon* 113 251–254. 10.5248/113.251

[B24] Goto B. T. Silva G. A. Assis D. M. A. Silva D. K. A. Souza R. G. Ferreira A. C. A. (2012). Intraornatosporaceae (Gigasporales), a new family with two new genera and two new species. *Mycotaxon* 119 117–132. 10.5248/119.117

[B25] Hammer Ø Harper D. A. T. Ryan P. D. (2001). PAST: paleontological statistics software package for education and data analysis. *Palaeontol. Electron.* 4 1–9.

[B26] Hart M. M. Reader R. J. (2002). Taxonomic basis for variation in the colonization strategy of arbuscular mycorrhizal fungi. *New Phytol.* 153 335–344. 10.1046/j.0028-646x.2001.00312.x

[B27] Holt B. G. Lessard J. P. Borregaard M. K. Fritz S. A. Araújo M. B. Dimitrov D. (2013). An update of Wallace’s zoogeographical regions of the world. *Science* 339 74–78. 23258408 10.1126/science.1228282

[B28] Hoorn C. Wesselingh F. P. ter Steege H. Bermudez M. A. Mora A. Sevink J. (2010). Amazonia through time: andean uplift, climate change, landscape evolution, and biodiversity. *Science* 330 927–931. 10.1126/science.1194585 21071659

[B29] International Culture Collection of Glomeromycota [CICG] (2020). *International Culture Collection of Glomeromycota.* Available online at: https://sites.google.com/site/cicgfma/home (Acessed November 17, 2020).

[B30] Jobim K. Blaszkowski J. Niezgoda P. Kozlowska A. Zubek S. Mleczko S. (2019). New sporocarpic taxa in the phylum *Glomeromycota*: *Sclerocarpum amazonicum* gen. et sp. nov. in the family Glomeraceae (*Glomerales*), and Diversispora sporocarpia sp. nov. in the Diversisporaceae (*Diversisporales*). *Mycol. Prog.* 18 369–384. 10.1007/s11557-018-01462-2

[B31] Jobim K. Oliveira B. I. S. Goto B. T. (2016). Checklist of the *Glomeromycota* in the Brazilian Savanna. *Mycotaxon* 131 1–13. 10.1007/978-3-319-23534-9_1

[B32] Jobim K. Vista X. M. Goto B. T. (2018). Updates on the knowledge of arbuscular mycorrhizal fungi (Glomeromycotina) in the Atlantic Forest biome – an example of very high species richness in Brazilian biomes. *Mycotaxon* 133 1–17.

[B33] Lugo M. A. Cabello M. N. (2002). Native arbuscular mycorrhizal fungi (AMF) from mountain grassland (Coìrdoba, Argentina) I. Seasonal variation of fungal spore diversity. *Mycologia* 94 579–586. 10.2307/3761709 21156531

[B34] Lugo M. A. Ferrero M. A. Menoyo E. Esteìvez M. C. Sinþeriz F. Anton A. M. (2008). Arbuscular mycorrhizal fungi and rhizospheric bacteria diversity along an altitudinal gradient in South American Puna grassland. *Microb. Ecol.* 55 705–713. 10.1007/s00248-007-9313-3 17912580

[B35] Maia L. C. Passos J. H. Silva J. A. Oehl F. Assis D. M. A. (2020). Species diversity of glomeromycota in brazilian biomes. *Sydowia* 72 181–205.

[B36] Moreira M. Baretta D. Tsai S. M. Gomes-da-Costa S. M. Cardoso E. J. B. N. (2007). Biodiversity and distribution of arbuscular mycorrhizal fungi in Araucaria angustifolia forest. *Sci. Agric.* 64 393–399. 10.1590/s0103-90162007000400010

[B37] Myers N. Mittermeier R. A. Mittermeier C. G. Fonseca G. A. B. Kent J. (2000). Biodiversity hotspots for conservation priorities. *Nature* 403 853–858. 10.1038/35002501 10706275

[B38] Olson D. M. Dinerstein E. Wikramanayake E. D. Burges N. D. Powell G. V. N. Underwood E. C. (2001). Terrestrial ecoregions of the world: a new map of life on Earth. *BioScience* 51 933–938. 10.1641/0006-3568(2001)051[0933:teotwa]2.0.co;2

[B39] Parenti L. R. Ebach M. C. (2009). *Comparative Biogeography—Discovering and Classifying Biogeographical Patterns of a Dynamic Earth.* Berkeley: Univ of California Press.

[B40] Pereira C. M. R. da Silva D. K. A. Ferreira A. C. A. Goto B. T. Maia L. C. (2014). Diversity of arbuscular mycorrhizal fungi in Atlantic forest areas under different land uses. *Agric. Ecosyst. Environ.* 185 245–252. 10.1016/j.agee.2014.01.005

[B41] Pontes J. S. Oehl F. Pereira C. D. Machado C. T. T. Coyne D. Silva D. K. A. (2017). Diversity of arbuscular mycorrhizal fungi in the Brazilian’s Cerrado and in soybean under conservation and conventional tillage. *Appl. Soil Ecol.* 117-118 178–189. 10.1016/j.apsoil.2017.04.023

[B42] Powell J. R. Bennett A. E. (2016). Unpredictable assembly of arbuscular mycorrhizal fungal communities. *Pedobiologia* 59 11–15. 10.1016/j.pedobi.2015.12.001

[B43] Prance G. T. (1973). Phytogeographical support for the theory of Pleistocene forest refuges in the Amazon basin, based on evidence from distribution patterns in *Caryocaraceae*, *Chrysobalanaceae*, *Dichapetalaceae* and *Lecythidaceae*. *Acta Amazon.* 3 5–28. 10.1590/1809-43921973033005

[B44] Redecker D. Schussler A. Stockinger H. Sturmer S. L. Morton J. B. Walker C. (2013). An evidence-based consensus for the classification of arbuscular mycorrhizal fungi (Glomeromycota). *Mycorrhiza* 23 515–531. 10.1007/s00572-013-0486-y 23558516

[B45] Remy W. Taylor T. N. Hass H. Kerp H. (1994). Four hundred-million-year- old vesicular arbuscularmycorrhizae. *Proc. Natl. Acad. Sci. U.S.A.* 91 11841–11843. 10.1073/pnas.91.25.11841 11607500 PMC45331

[B46] Schneider J. Stürmer S. L. Guilherme L. R. G. Moreira F. M. S. Soares C. R. F. S. (2013). Arbuscular mycorrhizal fungi in arsenic-contaminated areas in Brazil. *J. Hazard. Mater.* 262 1105–1115. 10.1016/j.jhazmat.2012.09.063 23102714

[B47] Schussler A. Mollenhauer D. Schnepf E. Kluge M. (1994). *Geosiphon pyriforme*, an endosymbiotic association of fungus and cyanobacteria: the spore structure resembles that of arbuscular mycorrhizal (AM) fungi. *Plant Biol.* 107 36–45. 10.1111/j.1438-8677.1994.tb00406.x

[B48] Silva I. R. Mello C. M. A. Neto R. A. F. Silva D. K. A. Melo A. L. Oehl F. (2014). Diversity of arbuscular mycorrhizal fungi along an environmental gradient in the Brazilian semiarid. *Appl. Soil Ecol.* 84 166–175. 10.1016/j.apsoil.2014.07.008

[B49] Siqueira J. O. Colozzi-Filho A. Oliveira E. (1989). Ocorrência de micorrizas vesicular-arbusculares em agro e ecossistemas do estado de Minas Gerais. *Pesq. Agropec. Bras.* 24 1499–1506.

[B50] Souza R. G. da Silva D. K. A. Mello C. M. A. Goto B. T. Silva F. S. B. Sampaio E. V. S. B. (2013). Arbuscular mycorrhizal fungi in revegetated mined dunes. *Land Degrad. Dev.* 24 147–155. 10.1002/ldr.1113

[B51] Stürmer S. L. Bellei M. M. (1994). Composition and seasonal variation of spore populations of arbuscular mycorrhizal fungi in dune soils on the island of Santa Catarina, Brazil. *Can. J. Bot.* 72 359–363. 10.1139/b94-048

[B52] Stürmer S. L. Bever J. D. Morton J. B. (2018). Biogeography of arbuscular mycorrhizal fungi (*Glomeromycota*): a phylogenetic perspective on species distribution patterns. *Mycorrhiza* 28 587–603. 10.1007/s00572-018-0864-6 30187122

[B53] Stürmer S. L. Siqueira J. O. (2011). Species richness and spore abundance of arbuscular mycorrhizal fungi across distinct land uses in Western Brazilian Amazon. *Mycorrhiza* 21 255–267. 10.1007/s00572-010-0330-6 20645112

[B54] Van der Heijden M. Klironomos J. Ursic M. Moutoglis P. Streitwolf-Engel R. Boller T. (1998). Mycorrhizal fungal diversity determines plant biodiversity, ecosystem variability and productivity. *Nature* 396 69–72. 10.1038/23932

[B55] Van der Heijden M. G. A. Bardgett R. D. van Straalen N. M. (2008). The unseen majority: soil microbes as drivers of plant diversity and productivity in terrestrial ecosystems. *Ecol. Lett.* 11 296–310. 10.1111/j.1461-0248.2007.01139.x 18047587

[B56] Velázquez M. S. Fabisik J. C. Abarca C. L. Allegrucci N. Cabello M. (2016a). Colonization dynamics of arbuscular mycorrhizal fungi (AMF) in *Ilex paraguariensis* crops: seasonality and influence of management practices. *J. King Saud. Univ.* 32 183–188. 10.1016/j.jksus.2018.03.017

[B57] Velázquez M. S. Stürmer S. L. Bruzone C. Fontenla S. Barrera M. Cabello M. (2016b). Occurrence of arbuscular mycorrhizal fungi in high altitude sites of the Patagonian Altoandina region in Nahuel Huapi National Park (Argentina). *Acta Bot. Bras.* 30 521–531. 10.1590/0102-33062016abb0223

[B58] Walker C. Trappe J. M. Schussler A. Hawksworth D. L. Cazares E. Elliott T. F. (2017). Proposal to conserve the name *Rhizophagus* with a conserved type (*Fungi*:*Glomeromycota*:Glomeraceae). *Taxon* 66 199–200. 10.12705/661.19

[B59] Wijayawardene N. N. Hyde K. D. Dai D. Q. Tang L. Z. Aptroot A. CastañedaRuiz R. F. (2020). Outline of *Fungi* and fungi-like taxa. *Mycosphere* 11 1060–1456. 10.5943/mycosphere/11/1/8 10549654

[B60] Zhang J. Wang F. Che R. Wang P. Liu H. Ji B. (2016). Precipitation shapes communities of arbuscular mycorrhizal fungi in Tibetan alpine steppe. *Sci. Rep.* 6:23488. 10.1038/srep23488 PMC4802204 27002188

