# Supplementary material for: The Glomeromycota in the Neotropics
Source: Front Microbiol. 2021 Jan 12;11:553679. doi: 10.3389/fmicb.2020.553679 (PMC7835493; doi:10.3389/fmicb.2020.553679)
Supplement: Supplementary file 2 [file Table_1.pdf]

**Supplementary Table for:**
**S.L. Stürmer and K. Kemmelmeier. The Glomeromycota in the Neotropics. Front. Microbiol.** ***11:553679. doi: 10.3389/fmicb.2020.553679***

Supplementary Table 1. Species of Glomeromycota and heterotypic synonym described from type-specimens originating in the Neotropics.

| Described as: | Changed to: | Authority | Reference |
|---|---|---|---|
| | | **Family Paraglomeraceae** | |
| *Pacispora boliviana* | *Paraglomus bolivianum* | (Sieverd. & Oehl) Oehl & G.A. Silva | Oehl, F. and E Sieverding 2004. J Appl Bot 78:72-82. |
| *Glomus brasilianum* | *Paraglomus brasilianum* | (Spain & J. Miranda) J.B. Morton & D. Redecker | Spain, J.L. and J.C. de Miranda. 1996. Mycotaxon 60:137-142. |
| *Paraglomus occidentale* | | Corazon-Guivin, G.A. Silva & Oehl | Corazon-Guivin, M. A., Cerna-Mendoza, A., Guerrero-Abad, J. C., Vallejos-Tapullima, A., Ríos-Ramírez, O., Vallejos-Torres, G., Sota-Ricaldi AM, Santos VM & Oehl, F. 2020. Sydowia 72: 85–94. |
| *Paraglomus pernambucanum* | | Oehl, C.M. Mello, Magna & G.A. Silva | Mello CMA, Silva GA, Assis DMA, Pontes JS, Ferreira ACA, Leão MPC, Vieira HEE, Maia LC, Oehl F. 2013. J Applied Bot and Food Quality 86:113-125. |
| | | **Family Ambisporaceae** | |
| *Acaulospora appendicula* | *Ambispora leptoticha* | (N.C. Schenck & G.S. Sm.) R.J. Bills & J.B. Morton | Schenck, N.C.; J.L. Spain; E. Sieverding and R.H. Howeler. 1984. Mycologia, 76:685-699. |
| | | **Family Archaeosporaceae** | |
| *Archaeospora ecuadoriana* | | A. Schüßler & C. Walker | Schussler A, Walker C. 2019. Mycorrhiza doi.org/10.1007/s00572-019-00913-2. |
| *Acaulospora myriocarpa* | *Archaeospora myriocarpa* | (Spain, Sieverd. & N.C. Schenck) Oehl, G.A. Silva, B.T. Goto & Sieverd. | Schenck, N.C.; J.L. Spain and E. Sieverding. 1986. Mycotaxon, 25:111-117. |
| *Entrophospora schenckii* | *Archaeospora schenckii* | (Sieverd. & S. Toro) C. Walker & Schüßler | Sieverding, E. and S. Toro. 1987. Mycotaxon, 28:209-214. |
| | | **Family Acaulosporaceae** | |

| | | | |
|---|---|---|---|
| *Entrophospora colombiana* | *Acaulospora colombiana* | (Spain & N.C. Schenck) Kaonongbua, J.B. Morton & Bever | Schenck, N.C.; Spain, J.L.; Sieverding, E.; Howeler, R,H. 1984. Mycologia. 76:685-699 |
| *Acaulospora aspera* | | Corazon-Guivin, Oehl & G.A. Silva | Corazon-Guivin MA, Cerna-Mendoza A, Guerrero-Abad JC, Vallejos-Tapullima A, Silva GA, Oehl F. 2019. J App Bot and Food Quality 92:250-257. |
| *Ambispora brasiliensis* | *Acaulospora brasiliensis* | (B.T. Goto, L.C. Maia & Oehl) C. Walker, M. Krüger & A. Schüßler | Goto BT, Maia LC, Oehl F. 2008. Mycotaxon 105:11-18. |
| *Acaulospora denticulata* | | Sieverd. & S. Toro | Sieverding, E. and S. Toro. 1987. Angewandte Botanik, 61:217-223. |
| *Acaulospora endographis* | | B.T. Goto | Goto BT, Pereira CMR, Nobre CP, Zatorre NP, Covacevich F, Berbara RLL, Maia LC. 2013. Mycotaxon 123:403-408. |
| *Acaulospora entreriana* | | M.S. Velázquez & Cabello | M.S. Velazquez, M. Cabell, G. Irrazabal and A. Godeas. 2008. Mycotaxon, 103:171-187. |
| *Acaulospora foveata* | | Trappe & Janos | Janos, D.P. and J.M. Trappe 1982. Mycotaxon 15:515-522 |
| *Acaulospora herrerae* | | Furrazola, B.T. Goto, G.A. Silva, Sieverd. & Oehl | Furrazola E, Goto BT, Silva GA, Torres-Arias Y, Morais T, Lima CEP, Ferreira ACA, Porto MCL, Maia LC, Sieverding E, Oehl F. 2013. Nova Hedwigia. 97:401-413 |
| *Acaulospora ignota* | | Błaszk., Góralska, Chwat & Goto | Błaszkowski, J; Chwat, G; Góralska, A. 2015. Mycological Progress. 14(4/18):1-11 |
| *Acaulospora longula* | | Spain & N.C. Schenck | Schenck, N.C.; J.L. Spain; E. Sieverding and R.H. Howeler. 1984. Mycologia, 76:685-699. |
| *Acaulospora mellea* | | Spain & N.C. Schenck | Schenck, N.C.; J.L. Spain; E. Sieverding and R.H. Howeler. 1984. Mycologia, 76:685-699. |
| *Acaulospora morrowiae* | | Spain & N.C. Schenck | Schenck, N.C.; J.L. Spain; E. Sieverding and R.H. Howeler. 1984. Mycologia, 76:685-699. |

| | | | |
|---|---|---|---|
| *Acaulospora papillosa* | | C.M.R. Pereira & Oehl | Pereira CMR, Maia LC, Sanchez-Castro I, Palenzuela J, Silva DKA, Sudová R, Kolarikova Z, Rydlova J, Ctvrtlijova M, Goto BT, Silva GA, Oehl F. 2016. Phytotaxa 260:14-24. |
| *Acaulospora reducta* | | Oehl, B.T. Goto & C.M.R. Pereira | Pereira CMR, Goto BT, Silva DKA, Ferreira ACA, Souza FA, Silva GA, Maia LC, Oehl F. 2015. Mycotaxon 130:983-9995. |
| *Acaulospora rehmii* | | Sieverd. & Toro | Sieverding, E.; Toro, S. 1987. Angewandte Botanik. 61:217-223 |
| *Acaulospora scrobiculata* | | Trappe | Trappe, J.M. 1977. Mycotaxon. 6(2):359-366 |
| *Acaulospora spinulifera* | | Oehl, V.M. Santos, J.S. Pontes & G.A. Silva | Pontes JS, Santos VM, Pereira CD, Silva GA, Maia LC, Oehl F. 2017. Nova Hedwigia, 105:219-229. |
| *Acaulospora splendida* | | Sieverd., Chaverri & I. Rojas | Sieverding, E.; Chaverri, A.; Rojas, I. 1988. Mycotaxon. 33:251-256 |
| *Acaulospora tuberculata* | | Janos & Trappe | Janos, D.P.; Trappe, J.M. 1982. Mycotaxon. 15:515-522 |
| | | **Family Diversisporaceae** | |
| *Corymbiglomus pacificum* | | Oehl, Medina, P. Cornejo, Sánchez-Castro, G.A. Silva & Palenz. | Medina J, Cornejo P, Borie F, Meier S, Palenzuela J, Vieira HEE, Ferreira ACA, Silva GA, Sánchez-Castro I, Oehl F. 2014. Mycotaxon 127:173-183. |
| *Diversispora varaderana* | | Błaszk., Chwat, Kovács & Góralska | Błaszkowski, J; Furrazola, E; Chwat, G; Góralska, A; Lukács, AF; Kovács, GM. 2015. Mycological Progress. 14(11/105):1-12 |
| *Glomus megalocarpum* | *Redeckera megalocarpum* | (D. Redecker) C. Walker & A. Schüßler | Redecker, D.; Raab, P.; Oehl, F.; Camacho, F.J.; Courtecuisse, R. 2007. Mycological Progress. 6(1):35-44 |

| | | | |
|---|---|---|---|
| *Glomus pulvinatum* | *Redeckera pulvinatum* | (Henn.) C. Walker & A. Schüßler | Thaxter, R. 1922. Proc. Amer. Acad. Sci., 57:291-350. |
| | | **Family Gigasporaceae** | |
| *Bulbospora minima* | | Oehl, Marinho, B.T. Goto & G.A. Silva | Marinho F, Silva GA, Ferreira ACA, Veras JSN, Sousa NMF, Goto BT, Maia LC, Oehl F. 2014. Sydowia 66:313-323. |
| *Cetraspora auronigra* | | Oehl, L.L. Lima, Kozovits, Magna & G.A. Silva | Lima LL, Kozovits AR, Assis DMA, Silva GA, Oehl F. 2014. Sydowia 66:299-308. |
| *Scutellospora biornata* | *Dentiscutata biornata* | (Spain, Sieverd. & S. Toro) Sieverd., F.A. de Souza & Oehl | Spain, J.L.; E. Sieverding and S. Toro. 1989. Mycotaxon, 35:219-227. |
| *Scutellospora cerradensis* | *Dentiscutata cerradensis* | (Spain & J. Miranda) Sieverd., F.A. de Souza & Oehl | Spain, J.L. and J.C. de Miranda. 1996. Mycotaxon 60:129-136. |
| *Dentiscutata colliculosa* | | B.T. Goto & Oehl | Goto, B.T.; da Silva, G.A.; Costa Maia, L.; Oehl, F. 2010. Nova Hedwigia. 90(3-4):383-393 |
| *Scutellospora savannicola* | *Dentiscutata savannicola* | (R.A. Herrera & Ferrer) C. Walker & A. Schussler | Ferrer R.L and R.A. Herrera. 1981. Rev. Jardin Bot Nacional Habana 1:43-66 |
| *Scutellospora scutata* | *Dentiscutata scutata* | (C. Walker & Dieder.) Sieverd., F.A. de Souza & Oehl | Walker, C. and C. Diederichs. 1989. Mycotaxon, 35:357-361. |
| *Fuscutata aurea* | | Oehl, C.M. Mello & G.A. Silva | Mello CMA, Silva GA, Vieira HEE, Silva IR, Maia LC, Oehl F. 2012. Nova Hedwigia 95:267-275. |
| *Racocetra intraornata* | *Intraornatospora intraornata* | (B.T. Goto & Oehl) B.T. Goto, Oehl & G.A. Silva | Spain, J.L.; E. Sieverding and N.C. Schenck. 1989. Mycotaxon, 34:667-677. |
| *Paradentiscutata bahiana* | | Oehl, Magna, B.T. Goto & G.A. Silva | Goto BT, Silva GA, Assis DMA, Silva DKA, Souza RG, Ferreira ACA, Jobim K, Mello CMA, Vieira HEE, Maia LC, Oehl F. 2012. Mycotaxon 119:117-132. |
| *Paradentiscutata maritima* | | B.T. Goto, D.K. Silva, Oehl & G.A. Silva | Goto BT, Silva GA, Assis DMA, Silva DKA, Souza RG, Ferreira ACA, Jobim K, Mello CMA, Vieira HEE, Maia LC, Oehl F. 2012. Mycotaxon 119:117-132. |

| | | | |
|---|---|---|---|
| *Racocetra crispa* | | F.A. de Souza, I. R. Silva, M.B. Barros-Barreto, B.T. Goto & Oehl | Sousa FA, Silva IR, Barreto MBBB, Oehl F, Goto BT, Maia LC. 2018. Mycological Progress do.org/10.1007/s11557-018-1410-9. |
| *Racocetra tropicana* | | Oehl, B.T. Goto & G.A. Silva | Goto BT, GA Silva, LC Maia, RG Souza, D Coyne, A Tchabi, L Lawouin,F Hountondji and F Oehl. 2011. Nova Hedwigia 92: 69–82. |
| *Gigaspora minuta* | *Racocetra minuta* | (Ferrer & R.A. Herrera) Oehl, F.A. Souza & Sieverd. | Oehl, F.; Souza, F.A. de; Sieverding, E. 2008. Mycotaxon. 106:311-360 |
| *Scutellospora alterata* | | Oehl, J.S. Pontes, Palenz., Sánchez-Castro & G.A. Silva | Pontes JS, Sánchez-Castro I, Palenzuela J, Maia LC, Silva GA, Oehl F. 2013. Mycotaxon 125:169-181. |
| *Scutellospora crenulata* | | R.A. Herrera-Peraza, Cuenca & C. Walker | Herrera-Peraza RA, G Cuenca & C Walker. 2001. Can J Bot 79: 674–678. |
| *Scutellospora pernambucana* | | Oehl, D.K Silva, N. Freitas, L.C. Maia | Silva DKA, Freitas NO, Cuenca G, Maia L, Oehl F. 2008. Mycotaxon 106:361-370. |
| *Scutellospora rubra* | | Stürmer & J.B. Morton | Stürmer, S.L. & Morton, J.B. 1999. Mycological Research 103:949-954. |
| *Scutellospora spinosissima* | | C. Walker & Cuenca | Walker C, Cuenca G, Sanchez F. 1998. Annals of Botany 82:721-725. |
| *Scutellospora striata* | | Cuenca & Herrera | Cuenca G, RA Herrera-Peraza. 2008. Mycotaxon, 105-79-87. |
| *Scutellospora tepuiensis* | | Furrazola & Cuenca | Andrade Z, Furrazola E, Cuenca G. 2017. Mycotaxon 132:9-18. |
| *Gigaspora tricalypta* | *Scutellospora tricalypta* | (R.A. Herrera & Ferrer) C. Walker & F.E. Sanders | Walker, C.; Sanders, F.E. 1986. Mycotaxon. 27:169-182 |
| | | **Family Pacisporaceae** | |
| *Glomus patagonicum* | *Pacispora patagonica* | (Novas & Fracchia) C. Walker, Vestberg & A. Schüßler | Novas, M.V.; Fracchia, S.; Menéndez, A.; Cabral, D.; Godeas, A. 2005. Nova Hedwigia. 80(3-4):533-539 |

| | | |
|---|---|---|
| | **Family Claroideoglomeraceae** | |
| *Claroideoglomus hanlinii* | Błaszk., Chwat & Góralska | Błaszkowski, J; Chwat, G; Góralska, A. 2015. Mycological Progress. 14(4/18):1-11 |
| | **Family Glomeraceae** | |
| *Funneliglomus sanmartinensis* | Corazon-Guivin, G.A. Silva & Oehl | Corazon-Guivin MA, Mendoza AC, Guerrero-Adab JC, Vallejos-Tapullima A, Carballar-Hernández S, Silva GA, Oehl F. 2019. Sydowia 71:17-24. |
| *Glomus brohultii* | R.A. Herrera, Ferrer & Sieverd. | Herrera-Peraza, R. A., Ferrer, R. L., & Sieverding, E. (2003). Journal of Applied Botany: 77(1/2), 37-40. |
| *Glomus crenatum* | Furrazola, R.L. Ferrer, R.A. Herrera & B.T. Goto | Furrazola, E.; Torres-Arias, Y.; Ferrer, R.L.; Herrera, R.A.; Berbara, R.L.L.; Goto, B.T. 2011. Mycotaxon. 116:143-149 |
| *Glomus cubense* | Y. Rodr. & Dalpé | Rodríguez, Y.; Dalpé, Y.; Séguin, S.; Fernández, K.; Fernández, F.; Rivera, R.A. 2011. Mycotaxon. 118:337-347 |
| *Glomus fuegianum* | (Speg.) Trappe & Gerd. | Thaxter, R. 1922. Proc. Amer. Acad. Arts Sci., 57:291-350. |
| *Glomus glomerulatum* | Sieverd. | Sieverding, E. 1987. Mycotaxon, 29:73-79. |
| *Glomus herrerae* | Torres-Arias, E. Furrazola & B.T. Goto | Torres-Arias, Y; Furrazola, E; Berbara, RLL; Jobim, K; Lima, JLR; Goto, BT. 2017. Current Research in Environmental & Applied Mycology 7(3):155-160 |
| *Glomus trufemii* | B.T. Goto, G. A. Silva & Oehl | Goto BT, Jardim JG, Silva GA, Furrazola E, Torres-Arias Y, Oehl F. 2012. Mycotaxon 120:1-9. |
| *Glomus segmentatum* | Trappe, Spooner & Ivory | Trappe, J.M. 1979. Transactions of the British Mycological Society. 73(2):361-362 |

| | | | |
|---|---|---|---|
| *Microkamienskia peruviana* | | Corazon-Guivin, G.A. Silva & Oehl | Corazon-Guivin MA, Cerna-Mendoza A, Guerrero-Abad JC, Vallejos-Tapullima A, Carballar-Hernández S, Silva GA, Oehl F. 2019. Nova Hedwigia 10.1127/nova-hedwigia/2019/0551 |
| *Nanoglomus plukenetiae* | | Corazon-Guivin, G.A. Silva & Oehl | Corazon-Guivin MA, Cerna-Mendoza A, Guerrero-Abad JC, Vallejos-Tapullima A, Carballar-Hernandez S, Silva GA, Oehl F. 2019. Mycological Progress 18:1395-1409. |
| *Rhizoglomus maiae* | | Błaszk., Piątek, Yorou, Zubek, Jobim, Niezgoda & B.T. Goto | Blaszkowski J, Niezgoda P, Piatek M, Magruno F, Malicka M, Zubek S, Mleczko P, Yorou NS, Jobim K, Vista XM, Lima JLR, Goto BT. 2019. Mycologia doi.org/10.1080/00275514.2019.1654637 |
| *Rhizoglomus variabile* | | Corazon-Guivin, Oehl & G.A. Silva | Song J et al. 2019. Sydowia 71:141-245. |
| *Glomus proliferum* | *Rhizophagus proliferus* | Dalpé & Declerck | Declerck, S.; Cranenbrouck, S.; Dalpé, Y.; Séguin, S.; Grandmougin-Ferjani, A.; Fontaine, J.; Sancholle, M. 2000. Mycologia. 92(8):1178-1187 |
| *Glomus manihotis* | *Rhizophagus manihotis* | (R.H. Howeler, Sieverd. & N.C. Schenck) C. Walker & A. Schüßler | Schenck, N.C.; J.L. Spain; E. Sieverding and R.H. Howeler. 1984. Mycologia, 76:685-699. |
| *Rhizophagus natalensis* | | Błaszk., Chwat & B.T. Goto | Blaszkowski J, Chwat G, Góralska A, Goto BT. 2014. Mycotaxon 129:97-108. |
| *Sclerocarpum amazonicum* | | Jobim, Błaszk., Niezgoda, Kozłowska & B.T. Goto | Jobim K, Blaszkowski J, Niezgoda P, Kozlowska A, Zubek S, Mleczko P, Chachula P, Ishikawa NK, Goto BT. 2019. Mycological Progress 18:369-384. |
| *Sclerocystis clavispora* | | Trappe | Trappe, J.M. 1977. Mycotaxon. 6(2):359-366 |
| *Glomus constrictum* | *Septoglomus constrictum* | (Trappe) Sieverd., G.A. Silva & Oehl | Trappe, J.M. 1977. Mycotaxon. 6(2):359-366 |

| | | | |
|---|---|---|---|
| *Septoglomus furcatum* | | Blaszk., Chwat, Kovács & Ryszka | Blaszkowski J, Chwat G, Kovács GM, Gáspár BK, Ryszka P, Orlowska E, Pagano MC, Araújo FS, Wubet T, Buscot F. 2013. Mycologia 105:670-680. |
| *Septoglomus titan* | | B.T. Goto & G.A. Silva | Goto BT, Araújo AF, Soares ACF, Ferreira ACA, Maia LC, Sousa CS, Silva GA. 2013. Mycotaxon 124:101-109. |
| | | **Heterotypic Synonym** | |
| *Fuscutata heterogama* | *Dentiscutata heterogama* | (T.H. Nicolson & Gerd.) Oehl, F.A. Souza, L.C. Maia & Sieverd. | Oehl F, Souza FA, Sieverding E. 2008. Mycotaxon. 106:311-360 |
| *Gigaspora ramisporophora* | *Gigaspora margarita* | Spain, Sieverd. & N.C. Schenck | Bentivenga SP, Morton JB. 1995. Mycologia 87:719-731 |
| *Endogone lignicola* | *Redeckera fulva* | Pat. | Patouillard NT. 1902. Bulletin de la Société Mycologique de France. 18(2):171-186 |
| *Endogone moelleri* | *Redeckera fulva* | Henn. | Hennings P. 1897. Hedwigia. 36:190-246 |
| *Ackermannia coccogena* | *Sclerocystis coremioides* | Pat. | Patouillard NT. 1902. Bulletin de la Société Mycologique de France. 18(2):171-186 |
| *Ackermannia dussi* | *Sclerocystis coremioides* | Pat. | Patouillard NT. 1902. Bulletin de la Société Mycologique de France. 18(2):171-186 |
