# Supplementary material for: The Glomeromycota in the Neotropics
Source: Front Microbiol. 2021 Jan 12;11:553679. doi: 10.3389/fmicb.2020.553679 (PMC7835493; doi:10.3389/fmicb.2020.553679)
Supplement: Supplementary file 3 [file Table_2.pdf]

Supplementary Table for:
S.L. Stürmer and K. Kemmelmeier. The Glomeromycota in the Neotropics. Front. Microbiol. ***11:553679. doi: 10.3389/fmicb.2020.553679***

Supplementary Table 2. Records of Glomeromycota from Neotropics according to taxonomic category and countries.

Country	Records	Species	Genera	Families	Orders
Argentina	716	77	20	9	4
Bolivia	70	20	11	6	3
Brazil	2836	182	32	11	4
Chile	207	58	19	9	4
Colombia	232	77	19	8	4
Equador	76	28	12	7	4
Peru	105	52	19	9	4
Venezuela	53	37	17	7	4
Belize	1	1	1	1	1
Costa Rica	85	42	18	7	3
Cuba	66	39	16	7	4
Guadaloupe	22	22	15	7	4
Guatemala	8	8	4	3	2
Honduras	12	12	7	5	3
Jamaica	8	3	2	1	1
Martinique	3	3	3	2	2
Mexico	463	87	21	10	4
Nicaragua	10	6	4	4	3
Panama	6	6	4	2	2
