# Supplementary material for: The Glomeromycota in the Neotropics
Source: Front Microbiol. 2021 Jan 12;11:553679. doi: 10.3389/fmicb.2020.553679 (PMC7835493; doi:10.3389/fmicb.2020.553679)
Supplement: Supplementary file 4 [file Table_3.pdf]

Supplementary Table for:
S.L. Stürmer and K. Kemmelmeier. The Glomeromycota in the Neotropics. Front. Microbiol. ***11:553679. doi: 10.3389/fmicb.2020.553679***

Supplementary Table 3. Number of species, genera and families of Glomeromycota in ecoregions of the Neotropics.

**Ecoregions**	**Species**	**Genera**	**Families**	**Country**
Alto Paraná Atlantic Forest	60	20	8	Brazil
Amazon-Orinoco-Southern Caribbean mangroves	15	7	3	Brazil
Araucaria moist forest	12	9	5	Brazil
Araya and Paria xeric scrub	19	14	6	Venezuela
Atlantic Coast restingas	36	15	6	Brazil
Bahamian-Antillean mangroves	32	17	8	Cuba, Guadeloupe
Bahia Coastal forests	17	12	6	Brazil
Bahia interior forests	59	17	8	Brazil
Bolivian montane dry forests	19	10	5	Bolivia
Brazilian Atlantic dry forests	10	5	4	Brazil
Caatinga	99	25	10	Brazil
Campos Rupestres montane savanna	33	11	6	Brazil
Cauca Valley montane forests	66	19	8	Colombia
Central American pine-oak forests	14	8	5	Mexico
Central Andean dry puna	15	9	5	Bolivia
Central Andean Puna	18	10	5	Bolivia
Cerrado	92	23	9	Brazil
Chilean Matorral	30	16	9	Chile
Costa Rican seasonal moist forests	1	1	1	Costa Rica
Cuban cactus scrub	1	1	1	Cuba
Cuban dry forests	24	13	6	Cuba
Cuban moist forests	2	2	1	Cuba
Cuban pine forests	12	8	3	Cuba
Dry Chaco	47	15	8	Argentina

Espinal	52	18	8	Argentina
Fernando de Noronha-Atol das Rocas moist forests	7	6	4	Brazil
Guianan savanna	21	9	5	Brazil
High Monte	1	1	1	Argentina
Humid Pampas	40	16	7	Argentina
Isthmian-Atlantic moist forests	5	3	2	Costa Rica, Panama
Jacurá-Solimões-Negro moist forests	25	14	7	Brazil
Magdalena Valley montane forests	2	2	2	Colombia
Magellanic subpolar forests	1	1	1	Argentina
Mesoamerican Gulf-Caribbean mangroves	22	12	6	Mexico
Oaxacan montane forests	24	12	5	Mexico
Pantanal	19	11	6	Brazil
Patagonian steppe	18	9	6	Argentina
Pernambuco coastal forests	33	14	7	Brazil
Pernambuco interior forests	48	18	6	Brazil
Petén-Veracruz moist forests	29	14	7	Mexico
Serra do Mar coastal forests	87	21	9	Brazil
Sierra de los Tuxtlas	39	18	10	Mexico
Sierra Madre de Chiapas moist forests	15	5	4	Mexico
Sierra Madre de Oaxaca pine-oak forests	4	4	3	Mexico
Sierra Madre del Sur pine-oak forests	7	5	3	Mexico
Southern Atlantic Brazilian mangroves	9	6	5	Brazil
Southern Pacific dry forests	35	15	8	Mexico
Southwest Amazon moist forests	22	10	6	Brazil
Talamancan montane forests	17	11	6	Costa Rica
Uatumã-Trombetas moist forests	50	18	9	Brazil
Uruguayan savanna	11	8	4	Brazil
Valdivian temperate forests	51	18	9	Argentina, Chile
